# Molecular role of non-exonic variants in *CALPAIN 10* gene in polycystic ovarian syndrome in Saudi women

**DOI:** 10.3389/fendo.2023.1303747

**Published:** 2023-12-27

**Authors:** Arwa A. Alageel, Amal F. Alshammary, Imran Ali Khan

**Affiliations:** Department of Clinical Laboratory Sciences, College of Applied Medical Sciences, King Saud University, Riyadh, Saudi Arabia

**Keywords:** polycystic ovarian syndrome, non-PCOS, *CALP10*, SNPs, Saudi women, diabetes, obesity

## Abstract

**Introduction:**

Non-diabetic women with polycystic ovarian syndrome (PCOS) often have abnormal insulin regulation. Calpain 10 (CALP10) is a biomarker of type 2 diabetes mellitus, with some of its single-nucleotide polymorphisms (SNPs) influencing PCOS development.

**Methods:**

In this case-control study on 90 women each with and without PCOS, we explored the molecular role of five CALP10 SNPs using biochemical parameters and Sanger sequencing analyses.

**Results:**

Different genetic models, genotypes, and allele frequencies were significantly associated with UCSNP-19 (rs3842570; p=0.01), UCSNP-44 (rs2975760; p=0.009), UCSNP-56 (rs2975762; p<0.0001), and UCSNP-63 (rs5030952; p=0.0003) in women with PCOS. The multiple logistic regression model showed a strong association of CALP10 SNPs with fasting blood glucose (p<0.001). ANOVA showed significant associations with various biochemical parameters such as FSH (p=0.0001) in UCSNP-19 (rs3842570), FI (p=0.002), TG (p=0.01) in UCSNP-56 (rs2975762) and FBG (p=0.001), FI (p=0.004), FSH (p=0.02) & LDLc (p=0.04) in UCSNP-63 (rs5030952) SNPs. Haplotype analysis also revealed significant associations between different combinations of alleles in the studied 5 SNPs in women with PCOS (p<0.05). Generalized multifactor dimensionality reduction analysis showed the best gene–gene interactions among the five SNPs in CALP10I (p<0.05). However, dendrogram and graphical depletion models found no strong association in women with PCOS.

**Conclusion:**

In conclusion, this study confirms rs3842570, rs2975760, rs2975767, and rs5030952 SNPs in CALP10 gene is associated in diagnosed PCOS women in the Saudi Arabia.

## 1 Introduction

Polycystic ovarian syndrome (PCOS) is a heterogeneous condition characterized by hyperandrogenism (HA), polycystic ovaries, and dysfunctional ovulation, and it is associated with metabolic problems such as insulin resistance (IR) and obesity (1). According to the World Health Organization, PCOS affected approximately 116 million women worldwide in 2012 (2). PCOS also contributed to a 56% increase in female infertility (3). Women diagnosed with PCOS have various reproductive, metabolic, cardiovascular, and psychological comorbidities (4). A combination of genetic and environmental factors can predict the development of PCOS in women (5), e.g., type 2 diabetes mellitus (T2DM), IR, obstructive sleep apnea, and elevated blood pressure (6).

The global prevalence of PCOS in the female population is 5–21% depending on the diagnostic criteria utilized (7). PCOS was first diagnosed in 1990 by the NICHD. In 2003, both ESHRE and ASRM updated the definition at Rotterdam (8). Although there is no cure or treatment for PCOS, various medications can alleviate the symptoms (9). PCOS may clinically manifest as HA, oligoanovulation, and polycystic ovary morphology (PCOM). Women with PCOS are categorized into four phenotypes: HA+OA+PCOM, phenotype-A; HA+OA, phenotype-B; HA+PCOM, phenotype-C; and OA+PCOM, phenotype D (10). IR appears to be a key factor PCOS development, with the ovary being only one among several organs affected (11). Nearly 44–70% of women diagnosed with PCOS have IR with endometrial IR and impaired glucose transport, leading to chronic low-grade inflammation, immune dysfunction modifications in the vascular uterus, elevated endometrial gene expression, and cellular abnormalities (12).

PCOS is influenced by multiple factors, including discrete genes, gene–gene interactions, and the environment (13). Genome-wide association studies (GWAS) identified 19 distinct genetic susceptibility loci in women with PCOS, with only 11 loci typically detected in both Han Chinese and European women. GWAS-identified loci only account for <10% of PCOS heredity, indicating that both environmental and non-environmental factors contribute to PCOS development (14). Single-nucleotide polymorphisms (SNPs) may reveal functional changes arising from variations in amino acids or gene expression regulation (15). The complexity of PCOS and the multiple genes implicated in its pathogenesis make genetics a valuable research tool (16). Cooper et al. raised doubts about the genetic basis of PCOS. Since then, many genes have been analyzed for possible links with this disorder (17). Based on linkage analysis, CALPAIN 10 (CALP10) was identified in Mexican Americans and was associated with modified CAPN10 expression (18). *CALP10* increases genetic susceptibility, particularly towards T2DM, which shares etiological elements with PCOS (19). SNPs in CALP10 have been linked to disorders of glucose metabolism and IR, both of which may influence susceptibility to PCOS. The SNPs UCSNP-19 (rs3842570), UCSNP-43 (rs3792267), UCSNP-44 (rs2975760), UCSNP-56 (rs2975762), and UCSNP-63 (rs5030952) have been shown to play a role in PCOS development (20, 21). Global studies have provided both positive and negative associations with all five SNPs in women of diverse ethnicities with PCOS; however, no molecular studies have documented a link between Saudi women with PCOS and CALP10 SNPs. The prevalence of PCOS is increasing in Saudi Arabia owing to the high prevalence of obesity. Therefore, this study was designed to determine the molecular relationship of these five *CALP10* SNPs with PCOS in Saudi women. This study aims to contribute novel insights into the genetic basis of PCOS susceptibility in this population. This research holds promise for advancing our understanding of PCOS development, potentially paving the way for improved diagnostic and therapeutic strategies tailored to Saudi women with PCOS.

## 2 Materials and methods

### 2.1 Study design and plan

This study was designed before ethical approval for enrollment (E-23-7917) from the Institutional Review Board of the College of Medicine at King Saud University and was in line with the Declaration of Helsinki. We enrolled women after obtaining signed informed consent.

In this case-control study, 90 PCOS women and 90 non-PCOS (controls) were selected from patients who visited the outpatient clinic of the Department of Obstetrics and Gynecology at King Khalid University Hospital. Based on a previous study, 90 women with PCOS and 90 non-PCOS women were selected (22). The inclusion criteria for women with PCOS were Saudi nationality and Rotterdam criteria (23). Rotterdam criteria include the presence of (i) polycystic ovaries, (ii) oligoanovulation, and (iii) HA. Saudi women who did not meet the Rotterdam criteria were excluded. Non-PCOS women were selected based on a single ovary, regular menstruation, no family or self-histories of PCOS, and non-Rotterdam criteria. Women without PCOS and those with a family history of irregular menstruation and multiple ovaries were excluded. Samples were collected for 365 days in 2021. The study protocol included women aged 18–40 years. All women (n=180) completed the questionnaire and confirmed the absence of any infection or autoimmune, chronic, or other diseases.

### 2.2 Sample collection and anthropometric measurements

In this study, Body Mass Index (BMI) was measured using weight in kilograms (kgs) and height in centimeters squared (m^2^). The equation for BMI was found to be kg/m^2^ and BMI was divided into normal BMI (<24.9 kg/m^2^), overweight (25.0–29.9 kg/m^2^), obesity (30.0–34.9 kg/m^2^), morbid obesity-I (35.0–39.9 kg/m^2^), and morbid obesity-II (≥40 kg/m^2^). All BMI values were recorded when the women visited the outpatient clinic and were diagnosed with PCOS or non-PCOS. Blood was collected from the women and stored in 360 plain vacutainers and 180 EDTA vacutainers. In each vacutainer, 1 mL, 1 mL, and 2 mL blood samples were collected from each woman, i.e., each woman gave 4 mL peripheral blood collected with two coagulants (1 mL + 1 mL) in plain tubes and 2 mL anticoagulant blood in EDTA tubes. The blood collected in the coagulant tubes was used to separate the serum by spinning at 4000 rpm for 10 min. Serum was extracted from the vacutainer tube, and peripheral blood in EDTA tubes was used for molecular analyses such as DNA isolation, PCR, and Sanger sequencing.

### 2.3 Serum studies

Fasting blood samples were drawn before a minimum of 1/3 day of overnight fasting. The same sample was used to measure FI. The remaining 1 mL of the sample was used to screen creatinine, FSH, TSH, LH, and TT levels. Additionally, TC, TG, HDLc, and LDLc were measured. Using serum samples, FBG, FI, creatinine, FSH, LH, TT, TC, TG and HDLc levels were measured using COBAS equipment and ROCHE kits. However, LDLc levels were calculated manually.

### 2.4 Molecular analysis of *CALP10* SNPs

EDTA blood was used to extract genomic DNA using the Qiagen DNA isolation Kit (Hilden, Germany). The quality and quantity of DNA were measured using a NanoDrop spectrophotometer at a 260/280 ratio. Genotyping was performed with PCR analysis in 50 µL reactions using Qiagen Hot Star Taq Master mix kit containing 10x buffer, 25 mM MgCl^2^, dNTPs, and 5 units of Taq DNA polymerase. Forward and reverse primers (10 pmol each) were synthesized by IRABIO (Hyderabad, India). Finally, 20 ng genomic DNA was used in a 50 µL reaction volume. Initial denaturation temperatures were optimized for each SNP: rs3842570 (68°C), rs3792267 (68°C), rs2975760 (60°C), rs2975762 (66°C), rs5030952 (66°C). This was followed by 35 cycles of denaturation at 95°C for 30 s; annealing between 60–68°C; and extension at 72°C for 45 s. Final extension was completed at 95°C for 5 min, followed by a final hold at 4°C. The cycling time of the five SNPs was between 1.23 and 1.35 h. All PCR products (187, 245, 240, 217, and 192 bp) were subjected to 2% agarose gel electrophoresis followed by ethidium bromide staining. The details of the SNP ID, primer sequences, methodology, and band sizes are shown in **Table 1**.

**Table 1:** Details of *CALP10* gene, primer sequences and band sizes along with methodology.

UCSNP ID	SNP ID	GENE LOCATION	FORWARD SEQUENCE	REVERSE SEQUENCE	PCR Size	Method	Band Sizes
UCSNP-19	rs3842570(Ins-Del)	Intron-6	AGACAGTGGGCTTTGACTCG	AGACAGTGGGCTTTGACTCG	187bp	PCR	Ins-187bp; Del-155bp
UCSNP-43	rs3792267 (G-A)	Intron-3	GCTGGCTGGTGACATCAGTGC	GCTGGCTGGTGACATCAGTGC	245bp	Sequencing	245bp
UCSNP-44	rs2975760 (T-C)	Intron-3	GATGTGGGCATCCATAGCTT	TGATCCCATGGTCTGTAGCA	240bp	Sequencing	240bp
UCSNP-56	rs2975762 (G-A)	Intron-4	AGGCCTCAGGCACACTGTAG	AGACAGTGGGCTTTGACTCG	217bp	Sequencing	217bp
UCSNP-63	rs5030952 (C-T)	3’UTR	AAGGGGGGCCAGGGCCTGACGGGGGTGGC	AGCACTCCCAGCTCCTGAT	192bp	PCR-RFLP	C-162/30bp; T-192bp

2Repeats, 155bp; 3Repeats, 187bp; Ins, Insertion and Del, Deletion.

### 2.5 Restriction enzyme and sanger sequencing analysis

**Figure 1:**
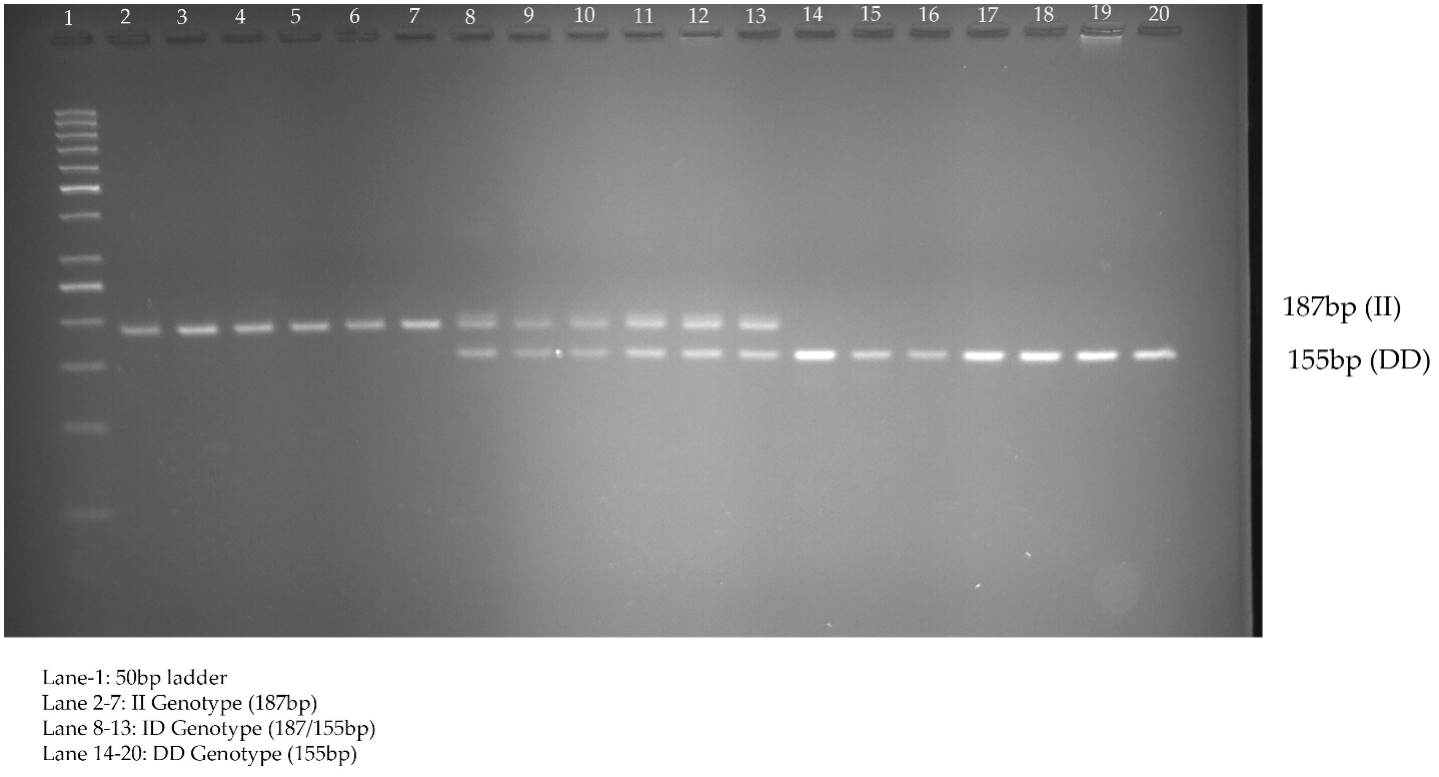
Genotyping of UCSNP-19 (rs3842570) variant studied in PCOS women through direct PCR analysis.

**Figure 2:**
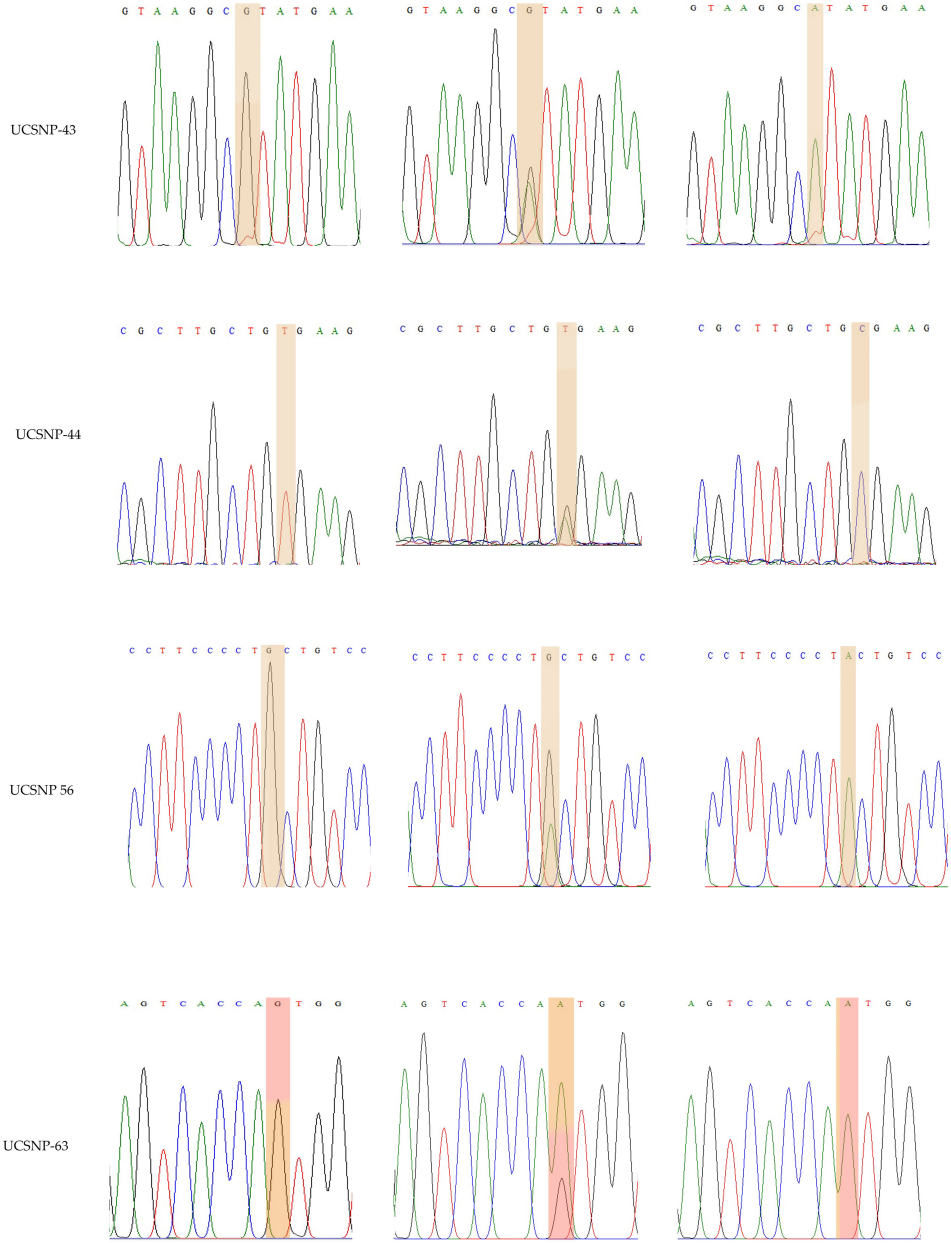
Representation of various traces of Sanger sequencing analysis for UCSNP-43, UCSNP-44, UCSNP56 and UCSNP-63 SNPs in *CALP 10* gene.

rs3842570 showed 187 bp as an insertion band and 155 bp as a deletion band. The insertion band comprised three repeats, and the deletion comprised two repeats. Each repeat contains a 32 bp sequence (**Figure 1**). rs5030952 was analyzed by PCR, followed by HhaI digestion for restriction fragment length polymorphism (RFLP) analysis. PCR products were digested with 5 U of HhaI enzyme in a 20 µL reaction volume containing 10x buffer, PCR product, and double distilled water; digestion was started with the final centrifugation for 30 s at 3000 rpm for 18 h at 37°C. Based on the presence of 162 and 30 bp bands confirmed as the CC genotype, the PCR product or TT genotype will have 192 bp and finally, 192, 162, and 30 bp were confirmed as the CT genotype or heterozygous genotype. The digested PCR products were separated by 3% agarose gel electrophoresis to confirm differences between the C and T alleles. For the remaining SNPs (rs3792267, rs2975760, and rs2975762), Sanger sequencing analysis was performed using a combination of specific primers and Big Dye sequencing using a Genetic Analyzer (Applied Biosystems) for multicapillary sequencing analysis. The four nucleotides are represented by different colors: A=green, T=red, G=black, and C=blue. The presence of a single peak indicates homozygosity, whereas the presence of two peaks represents a heterozygous peak that overlaps. UCSNP-19 encodes the genotypes II, ID, and DD. Other SNPs (UCSNP-43 (G-A), UCSNP–44 (T-C), UCSNP56 (G-A), and UCSNP-63 (C-T)) represent particular nucleotide changes (**Table 1**). Sequencing was performed bidirectionally, and finally, ABI, fasta, and PDF files were received. The sequencing files were analyzed and using BioEdit. PDF files were used as cross-references to reconfirm the analysis. Complete molecular analysis was performed in the G-141 laboratory at KSU, and Sanger sequencing was performed outside the laboratory. To validate the rs3842570 and rs5030952 SNPs, Sanger sequencing was repeated for 10% of the PCR products; the results confirmed 100% accuracy of the PCR and PCR-RFLP analyses (**Figure 2**). This indicated that the quality control processed in the G-141 laboratory results matched 100% with the validation of the Sanger sequencing results. Finally, the concordance rate was considered to be above 99% among 10% of the random known samples.

### 2.6 Statistical analysis

Biochemical, clinical, anthropometric, and genotypic data were recorded in Microsoft Excel. Categorical or nominal variables were documented as frequencies and percentages (%); numerical variables are indicated as mean ± standard deviation. Continuous data were analyzed using the unpaired *t*-test; numerical data were analyzed using Fisher’s exact test or χ^2^-square tests. This study conducted a Shapiro-Wilk test of normality to detect the data as normal distribution. Mann-Whitney U test was applied to analyze the statistical differences between the two groups (PCOS vs non-PCOS women). Using SNPStat software (24), we calculated (i) HWE; (ii) genotype and allele frequencies with 95%CIs, ORs, and p-values; and (iii) haplotypes separately for PCOS and non-PCOS women. A multiple linear regression model was used to test the independent predictors in women with PCOS, along with the SNPs present in CALP10, using SPSS software (25) (version 27.0). ANOVA with logarithmic adjustment of non-normally distributed data and *post hoc* correction for multiple comparisons was used to compare the three genotype groups in CALP10 using Jamovi software (24). Haploview (version 4.2) software (26) was used to test the LD in the studied women along with the coefficient (D’). Gene–gene interactions, dendrograms, and graphical depletion models were studied using the GMDR model with five SNPs (27). Statistical significance was set at p ≤ 0.05.

## 3 Results

### 3.1 Demographic details of PCOS and non-PCOS women using Shapirowilk and Mann-Whitney U test

We selected 180 women (90 women each with and without PCOS) after screening according to Rotterdam criteria. The clinical and demographic features of both groups are shown in **Table 2** for Shapiro Wilk test and **Table 3** includes Mann-Whitney U tests. Non-PCOS women served as controls for comparison with women with PCOS. Age (31.57 ± 6.72 vs. 30.80 ± 5.58; p=0.230), weight (77.41 ± 12.04 vs. 73.60 ± 11.47; p=0.829), body mass index (BMI; 30.67 ± 4.62 vs. 29.03 ± 4.84; p=0.710), and thyroid-stimulating hormone (TSH; 2.41 ± 0.31 vs. 2.23 ± 0.85; p=0.394) levels and LH/FSH ratio (1.18 ± 0.72 vs. 1.40 ± 0.88; p<0.001) were higher in non-PCOS than in PCOS women. Other parameters such as fasting blood glucose (FBG; 5.03 ± 0.88 vs. 4.77 ± 0.73; p<0.001), fasting insulin (FI; 11.03 ± 6.19 vs. 8.63 ± 1.05; p<0.001), HOMA-IR (2.60 ± 2.02 vs. 1.83 ± 0.35; p<0.001) creatinine (53.48 ± 13.49 vs. 46.86 ± 17.82; p<0.001), total testosterone (TT; 1.87 ± 0.88 vs. 0.86 ± 0.24; p<0.001), follicle-stimulating hormone (FSH; 6.84 ± 2.81 vs. 6.08 ± 2.50; p<0.001), luteinizing hormone (LH; 7.38 ± 4.41 vs. 7.07 ± 2.38; p<0.001), and triglyceride levels (TG; 1.81 ± 1.12 vs. 1.64 ± 0.91; p<0.001), high-density lipoprotein cholesterol (HDLc; 0.62 ± 0.23 vs. 0.48 ± 0.15; p=0.024), family history of PCOS (p<0.0001), and other family histories (p=0.047) were higher in PCOS women and were significantly associated. However, height (159.03 ± 5.23 vs. 158.91 ± 6.82; p=0.166), total cholesterol (TC; 5.06 ± 1.09 vs. 3.10 ± 0.39; p=0.104), and low-density lipoprotein cholesterol (LDLc; 3.62 ± 0.93 vs. 3.20 ± 0.55; p=0.076) were found to be high in PCOS but were not associated when compared with non-PCOS women. The **Table 3** analysis of Mann Whitney U test confirmed BMI (p=0.026), FBG (p=0.028), creatinine (p<0.001), FSH (p=0.026), Total testosterone (p<0.001), TC (p<0.001), HDLc (p<0.001) and LDLc (p<0.001) levels were associated.

**Table 2:** Anthropometric and biochemical profiles of PCOS and non-PCOS women.

Phenotypic characteristics	PCOS women (n=90)	Non-PCOS women (n=90)	Shapiro Wilk		P value
			Statistic	df	
Age (Years)	30.80 ± 5.58	31.57 ± 6.72	0.967	89	0.230
Weight (cms)	73.60 ± 11.47	77.41 ± 12.04	0.991	89	0.829
Height (cms)	159.03 ± 5.23	158.91 ± 6.82	0.979	89	0.166
BMI (kg/m^2^)	29.03 ± 4.84	30.67 ± 4.62	0.990	89	0.710
FBG (mmol/L)	5.03 ± 0.88	4.77 ± 0.73	0.885	89	<0.001
FI (µIU/mL)	11.03 ± 6.19	8.63 ± 1.05	0.740	89	<0.001
HOMA-IR	2.60 ± 2.03	1.83 ± 0.35	0.664	89	<0.001
Creatinine (mcmol/L)	53.48 ± 13.49	46.86 ± 17.82	0.908	89	<0.001
FSH (IU/mL)	6.84 ± 2.81	6.08 ± 2.50	0.758	89	<0.001
LH (IU/mL)	7.38 ± 4.41	7.07 ± 2.38	0.897	89	<0.001
TSH (IU/mL)	2.23 ± 0.85	2.41 ± 0.31	0.985	89	0.394
LH/FSH ratio (IU/mL)	1.18 ± 0.72	1.40 ± 0.88	0.922	89	<0.001
Total Testosterone (nmol/L)	1.87 ± 0.88	0.86 ± 0.24	0.932	89	<0.001
TC (mmol/L)	5.06 ± 1.09	3.10 ± 0.39	0.976	89	0.104
TG (mmol/L)	1.81 ± 1.12	1.64 ± 0.91	0.832	89	<0.001
HDLc (mmol/L)	0.62 ± 0.23	0.48 ± 0.15	0.967	89	0.024
LDLc (mmol/L)	3.62 ± 0.93	3.20 ± 0.55	0.974	89	0.076
Family history of PCOS	22 (24.44%)	0 (0%)	NA	NA	<0.0001
Other family Histories	30 (33.33%)	26 (28.88%)	NA	NA	0.047

BMI, Body Mass Index; FBG, Fasting Blood Glucose;FI, Fasting Insulin;HOMA-IR, Homeostatstic Model Assessment for Insulin Resistance;FSH, Follicle stimulating hormone;LH, Leutinizing Hormone;TSH, Thyroid stimulating hormone;TC, Total cholesterole;TG, Triglycerides;HDLc, High Density Lipoprotein cholesterol and LDLc, Low Density lipoprotein cholesterol. NA, Not applicable.

**Table 3:** Between group comparison for the outcome variables, using a non-parametric Mann-Whitney U test (95% CI, 2-tailed).

Phenotypic characteristics (N=90/group)	Mean Rank	Sum of Ranks	Mann-Whitney U test		
			U-Statistic	Z-score	P-value (2-tailed)
Age Years)	87.04	7833.50	3738.500	-0.893	0.372
Weight (cms)	83.06	7475.50	3380.500	-1.916	0.055
Height (cms)	92.28	8305.00	3890.000	-0.458	0.647
BMI (kg/m^2^)	81.84	7366.00	3271.000	-2.229	0.026
FBG (mmol/L)	99.02	8911.50	3283.500	-2.193	0.028
FI (µIU/mL)	90.49	8054.00	3961.000	-0.127	0.899
HOMA-IR	96.03	8547.00	3468.000	-1.549	0.121
Creatinine (mcmol/L)	108.39	9755.50	2439.500	-4.609	<0.001
FSH (IU/mL)	99.14	8923.00	3272.000	-2.226	0.026
LH (IU/mL)	87.74	7897.00	3802.000	-0.710	0.478
TSH (IU/mL)	94.85	8536.50	3658.500	-1.120	0.263
LH/FSH ratio (IU/mL)	83.88	7549.00	3454.000	-1.705	0.088
Total Testosterone (nmol/L)	126.86	11417.50	777.500	-9.363	<0.001
TC (mmol/L)	131.49	11834.00	361.000	-10.554	<0.001
TG (mmol/L)	94.03	8463.00	3723.000	-0.910	0.363
HDLc (mmol/L)	107.15	9643.50	2551.500	-4.287	<0.001
LDLc (mmol/L)	103.78	9340.00	2855.000	-3.419	<0.001

BMI, Body Mass Index; FBG, Fasting Blood Glucose; FI, Fasting Insulin; HOMA-IR, Homeostatic Model of Assessment for Insulin Resistance; FSH, Follicle Stimulating Hormone; LH, Leutinizing Hormone; TSH, Thyroid Stimulating Hormone; TC, Total Cholesterol; TG, Triglycerides;HDLc, High Density Lipoprotein Cholesterol and LDLc, Low Density Lipoprotein Cholesterol.

### 3.2 Hardy–Weinberg equilibrium analysis

The genotypes of the five SNPs in the PCOS and non-PCOS groups were found by HWE analysis based on χ^2^-test results, confirming that the selected PCOS cases were representative of the population (**Table 4**). The HWE test showed that the distribution of the allele frequencies of rs3842570 (χ^2 =^ 2.13/p=0.14) and rs38425760 (χ^2 =^ 0.62/p=0.42), was consistent in women with PCOS, and that rs3792267 (χ^2 =^ 6.16/p=0.01), rs2975762 (χ^2 =^ 35.41/p<0.0001) and rs5030952 (χ^2 =^ 8.66/p=0.003) SNPs were consistent in non-PCOS women. The overall analysis confirmed that the included population was representative of the entire population for genetic analysis.

**Table 4:** The primary information on genetic SNPs in the *CALP10* gene and HWE analysis.

Gene	Genotyped SNPs	Chromosome Position	Minor Allele	PCOS (MAF)	Controls (MAF)	PCOS (χ^2^/P)	Non-PCOS (/χ^2^P)	Genotyping Value (%)
CALP10	rs3842570	2: 240594825-876	Deletion	62.2%	47.8%	0.26/0.60	2.13/0.14	100%
CALP10	rs3792267	2: 240591757	A allele	20%	12.8%	0.01/0.91	6.16/0.01	100%
CALP10	rs2975760	2: 240591746	C allele	24.4%	11.7%	4.27/0.03	0.62/0.42	100%
CALP10	rs2975762	2: 2405922320	A allele	53.9%	23.3%	17.51/0.00002	35.41/<0.0001	100%
CALP10	rs5030952	2: 240603286	T allele	16.1%	3.3%	13.23/0.0002	8.66/0.003	100%

CALP10, CALPAIN10; SNPs, Single Nucleotide Polymorphisms and MAF, Minor Allele Frequencies; χ2, chi-square value.

### 3.3 Genotyping analysis for intronic SNPs in *CALP10*

**Table 5** shows the genotypes identified between PCOS cases and non-PCOS controls among five different genotypes: rs3842570 (UCSNP-19), rs3792267 (UCSNP-43), rs2975760 (UCSNP-44), UCSNP56 (rs2975762), and UCSNP-63 (rs5030952). UCSNP-19 (rs3842570) is an insertion (I)-deletion (D) (ID) SNP in *CALP10*. Genotype II includes 187 bp, which indicates triplet repeats of 32 bp, while genotype DD contains 155 bp, which indicates double repeats of 32 bp (**Figure 1**). In women with PCOS, the II, ID, and DD genotypes were 15.6%, 44.4%, and 40%, and in women without PCOS, 31.1%, 42.2%, and 26.7%, respectively. Statistical association was found only with the dominant model (ID+DD vs. II: OR, 2.45 (95%CI: 1.18–5.05); p=0.01]]. No significant difference was found between genotypes and other genetic models (ID vs. II: OR, 2.11 (95%CI: 0.96–4.59); p=0.02; DD vs. II: OR, 1.71 (95%CI: 0.78–3.74); p=0.07; DD+II vs. ID: OR, 0.91 (95%CI: 0.50–1.64); p=0.76 and DD vs. II+ID: OR, 1.83 (95%CI: 0.97–3.44); p=0.057). The combination of ID and DD genotypes confirmed 2.4 times higher risk associated with PCOS. We did not observe any significant association between UCSNP-43 (rs3792267) and PCOS. The frequencies of the GG, GA, and AA genotypes in the PCOS cases were 66.8%, 26.7%, and 6.7%, and in non-PCOS, 76.7%, 21.1%, and 2.2%, respectively. None of the genotypes (GA vs. GG: OR, 1.45 (95%CI: 0.72–2.91); p=0.20; AA vs. GG: OR, 3.45 (95%CI: 0.67–17.74); p=0.11) or genetic models (GA+AA vs. GG: OR, 1.64 (95%CI: 0.82–3.16); p=0.13; AA+GG vs. GA: OR, 0.73 (95%CI: 0.36–1.46); p=0.38; AA vs. GA+GG: OR, 3.14 (95%CI: 0.61–16.01); p=0.14) showed significant association. Hence, rs3792267 is not associated with PCOS in Saudi women.

**Table 5:** Genotype frequencies and genetic models for PCOS and non-PCOS women in all 5 SNPs present in *CALP10* gene.

Gene (rs number)	Genotypes	PCOS (n=90)	Non-PCOS (n=90)	OR (95%CI) and P Value
*CALP10 (*rs3842570*)*	II (Wild type)	14 (15.6%)	28 (31.1%)	1.00
	ID (Heterozygous Mutant)	40 (44.4%)	38 (42.2%)	OR-2.11 (0.96-4.59); p=0.02
	DD (Homozygous Mutant)	36 (40.0%)	24 (26.7%)	OR-1.71 (0.78-3.74); p=0.17
	ID+DD Vs II (Dominant Model)	76 (84.4%)	62 (68.9%)	OR-2.45 (1.18-5.05); p=0.01
	DD+II Vs ID (SD/CD)	50 (55.6%)	52 (57.8%)	OR-0.91 (0.50-1.64); p=0.76
	DD Vs ID+II (Recessive Model)	36 (40.0%)	24 (26.7%)	OR-1.83 (0.97-3.44); p=0.057
*CALP10 (*rs3792267*)*	GG (Wild type)	60 (66.8%)	69 (76.7%)	1.00
	GA (Heterozygous Mutant)	24 (26.7%)	19 (21.1%)	OR-1.45 (0.72-2.91); p=0.29
	AA (Homozygous Mutant)	06 (6.7%)	02 (2.2%)	OR-3.45 (0.67-17.74); p=0.11
	GA+AA Vs GG (Dominant)	30 (33.4%)	21 (23.3%)	OR-1.64 (0.82-3.16); p=0.13
	AA+GG Vs GA (SD/CD)	66 (73.3%)	71 (78.9%)	OR-0.73 (0.36-1.46); p=0.38
	AA Vs GA+GG (Recessive)	06 (6.7%)	02 (2.2%)	OR-3.14 (0.61-16.01); p=0.14
*CALP10 (*rs2975760*)*	TT (Wild type)	55 (61.1%)	71 (78.9%)	1.00
	TC (Heterozygous Mutant)	26 (28.9%)	17 (18.9%)	OR-1.97 (0.97-3.99); p=0.056
	CC (Homozygous Mutant)	09 (10%)	02 (2.2%)	OR-5.81 (1.21-27.98); p=0.01
	TC+CC Vs TT (Dominant Model)	35 (38.9%)	19 (21.1%)	OR-2.37 (1.23-4.61); p=0.009
	CC+TT Vs TC (SD/CD)	64 (71.1%)	73 (81.1%)	OR-0.57 (0.28-1.15); p=0.11
	CC Vs TC+TT (Recessive Model)	09 (10%)	02 (2.2%)	OR-4.88 (1.02-23.3); p=0.02
*CALP10* (rs2975762)	GG (Wild type)	29 (32.2%)	63 (70.0%)	1.00
	GA (Heterozygous Mutant)	25 (27.8%)	12 (13.3%)	OR-4.52 (2.01-10.24); p=0.0001
	AA (Homozygous Mutant)	36 (40.0%)	15 (16.7%)	OR-5.21 (2.47-10.98); p<0.0001
	GA+AA Vs GG(Dominant)	61 (67.8%)	27 (30.0%)	OR-4.91 (2.61-9.23); p<0.0001
	AA+GG Vs GA (SD/CD)	65 (72.2%)	78 (86.7%)	OR-0.41 (0.18-0.86); p=0.01
	AA Vs GA+GG (Recessive)	36 (40.0%)	15 (16.7%)	OR-3.33 (1.67-6.68); p=0.0005
*CALP10* (rs5030952)	CC (Wild type)	68 (75.5%)	85 (94.5%)	1.00
	CT (Heterozygous Mutant)	15 (16.7%)	04 (4.4%)	OR-4.68 (1.48-14.78); p=0.004
	TT (Homozygous Mutant)	07 (7.8%)	01 (1.1%)	OR-8.75 (1.05-72.85); p=0.01
	CT+TT Vs CC (Dominant Model)	22 (24.4%)	05 (5.6%)	OR-5.51 (1.98-15.28); p=0.0003
	TT+CC Vs CT (SD/CD)	75 (83.3%)	86 (95.6%)	OR-0.69 (0.18-2.69); p=0.59
	TT Vs CT+CC (Recessive Model)	07 (7.8%)	01 (1.1%)	OR-7.51 (0.90-62.31); p=0.03

SD-Semi Dominant/CD-co-dominant models; OR-Odds Ratio and 95%CI, Confidence Intervals.

The TT, TC, and CC genotype distribution for UCSNP-44 (rs2975760) in PCOS and non-PCOS subjects was 61.1%, 28.9%, 10%, and 78.9%, 18.9%, and 2.2%, respectively. Genotype analysis (TC vs. TT: OR, 1.97 (95%CI: 0.97–3.99); p=0.056; CC vs. TT: OR, 5.81 (95%CI: 1.21–27.98); p=0.01)) confirmed a positive association with dominant (TC+CC vs. TT: OR, 2.37 (95%CI: 1.23–4.61); p=0.009) and recessive models (CC vs. TC+TT: OR, 4.88 (95%CI: 1.02–23.3); p=0.02). However, the co-dominant model CC+TT vs TC: OR-0.57 (95%CI: 0.28-1.15); p=0.11) was not associated (p=0.11).

UCSNP56 (rs2975762) was significantly associated with PCOS women among genotypes (GA vs. GG: OR, 4.52 (95%CI: 2.01–10.24); p=0.0001; vs. AA vs. GG: OR, 5.21 (95%CI: 2.47–10.98); p<0.0001) and dominant (GA+AA vs. GG: OR, 4.91 (95%CI: 2.61-9.23); p<0.0001) and recessive models (GA vs. GG: OR, 3.33 (95%CI: 1.67–6.68); p=0.0005). The co-dominant model (AA+GG vs GA: OR-0.41 (95%CI: 0.18-0.86); p=0.01) was not associated. The overall prevalence of GG, GA, and AA genotypes in patients with PCOS was 32.2%, 27.8%, and 40%, and for non-PCOS, 70%,13.3%, and 16.7%, respectively.

UCSNP-63 (rs5030952) was the final SNP studied. The CC, CT, and TT genotypes present in both PCOS (75.5%, 16.7%, 7.8%) and non-PCOS (94.5%, 4.4%, 1.1%) subjects were strongly associated with both the genotypes (CT vs. CC: OR, 4.68 (95%CI: 1.48–14.78); p=0.004; TT vs. CC: OR, 8.75 (95%CI: 1.05–72.85); p=0.0003). Among genetic models, only the dominant model (TT+CT vs. CC: OR, 5.51 (95%CI: 1.98–15.28); p=0.0003) was associated. and other models such as Recessive: TT vs CT+CC: OR-0.69 (95%CI: 0.18-2.69); p=0.59) and co-dominant models (TT+CC vs CT: OR-7.51 (95%CI: 0.90-62.31); p=0.03), negative association was documented.

### 3.4 Overall allele frequency analysis in five *CALP10* SNPs

Allele frequencies in PCOS and non-PCOS subjects are shown in **Table 6**. Among the five CALP10 SNPs studied, 80% differed significantly between the PCOS and non-PCOS groups. D allele frequency in UCSNP-19 (rs3842570) was 62.2% and 47.8% in cases and controls, and 52.2% and 37.8% in controls and cases of the recruited women. The allelic association was documented [D vs. I: OR, 1.81 (95%CI: 1.18–2.74); p=0.005]. The allele frequencies in UCSNP-43 (rs3792267) were not statistically associated due to the presence of 20% and 12.8% of A alleles and 80% and 87.2% of G alleles in both PCOS and non-PCOS subjects [A vs. G: OR, 1.71 (95%CI: 0.96–3.11); p=0.06]. The remaining SNPs, UCSNP-44 (rs2975760), UCSNP 56 (rs2975762), and UCSNP-63 (rs5030952), were significantly associated with PCOS and non-PCOS groups. The minor allele frequency (MAF) of the C allele was 24.4% in PCOS women and 11.7% in non-PCOS women [C vs. T: OR, 2.45 (95%CI: 1.39–4.32); p=0.01]. The MAF of the A allele in UCSNP56 was high in PCOS women (53.9%) and 23.3% in non-PCOS subjects [A vs. G: OR, 3.84 (95%CI: 2.44–6.04); p<0.0001. UCSNP-63 had a high T allele frequency in PCOS women (16.1%) and only 3.3% in non-PCOS women [T vs. C: OR, 5.57 (95%CI: 2.25–13.78); p=0.0004].

**Table 6:** Genetic Variants of 5 SNPs present in CALP10 gene and allele frequencies in PCOS and non-PCOS women.

Gene (rs number)	Alleles	PCOS (n=90)	Non-PCOS (n=110)	OR (95%CI) and P Value
*CALP10 (*rs3842570*)*	I	68 (37.8%)	94 (52.2%)	Reference
	D	112 (62.2%)	86 (47.8%)	OR-1.81 (1.18-2.74); p=0.005
*CALP10 (*rs3792267*)*	G	144 (80%)	157 (87.2%)	Reference
	A	36 (20%)	23 (12.8%)	OR-1.71 (0.96-3.11); p=0.06
*CALP10 (*rs2975760*)*	T	136 (75.6%)	159 (88.3%)	Reference
	C	44 (24.4%)	21 (11.7%)	OR-2.45 (1.39-4.32); p=0.001
*CALP10 (*rs2975762*)*	G	83 (46.1%)	138 (76.7%)	Reference
	A	97 (53.9%)	42 (23.3%)	OR-3.84 (2.44-6.04); p<0.0001
*CALP10 (*rs5030952*)*	C	151 (83.9%)	174 (96.7%)	Reference
	T	29 (16.1%)	06 (3.3%)	OR-5.57 (2.25-13.78); p=0.0004

^OR-Odds Ratio and 95%CI= Confidence Intervals^.

### 3.5 Multiple linear regression model

The dependent and independent variables in the multiple linear regression model are shown in **Table 7**. The dependent variables were age, weight, BMI, FBG, FI, creatinine, FSH, LH, TSH, TT, TC, TG, HDL-c, and LDL-c levels. The rs3842570, rs3792267, rs2975760, rs2975762, and rs5030952 SNPs were considered these as dependent variables. The genotypes of the independent variables were recorded as 1 for homozygous normal variants, 2 for heterozygous variants, and 3 for homozygous variants. The analysis performed with 14 dependent variables and five independent variables showed that FBG levels were significantly associated with PCOS (p<0.001); the remaining 13 variables showed non-significant associations (p>0.05).

**Table 7:** Analysis of Multiple linear regression model in SNPs present in *CALP10* gene and PCOS covariates.

Covariates	R-value^a^	Adjusted R Square value	Standardized β-coefficient for rs3842570	Standardized β-coefficient for rs3792267	Standardized β-coefficient for rs2975760	Standardized β-coefficient for rs2975762	Standardized β-coefficient for rs5030952	F	p value^b^
Age	0.160	-0.032	0.123	-0.114	-0.028	0.009	0.036	0.443	0.817
Weight	0.180	-0.025	-0.002	-0.080	-0.152	-0.004	-0.100	0.560	0.730
BMI	0.216	-0.010	0.023	-0.154	-0.142	-0.028	-0.106	0.821	0.538
FBG	0.507	0.213	-0.007	-0.148	-0.251	-0.002	0.397	5.809	<0.001
FI	0.296	0.033	-0.030	-0.098	-0.034	0.052	0.262	1.599	0.170
CREATININE	0.192	-0.020	0.130	-0.023	-0.143	0.051	-0.008	0.646	0.665
FSH	0.087	-0.052	-0.004	-0.045	0.012	0.001	0.072	0.128	0.986
LH	0.223	-0.007	0.183	-0.094	-0.079	-0.036	-0.069	0.876	0.501
TSH	0.224	-0.006	-0.112	-0.100	-0.121	-0.093	-0.057	0.890	0.492
TT	0.232	-0.002	-0.167	-0.064	0.090	0.072	0.049	0.958	0.449
TC	0.214	-0.011	-0.030	-0.063	-0.001	-0.172	-0.113	0.810	0.546
TG	0.222	-0.007	0.037	0.058	0.064	-0.135	0.179	0.873	0.503
HDLc	0.148	-0.036	-0.103	0.009	-0.053	-0.017	-0.107	0.379	0.862
LDLc	0.258	0.011	-0.031	-0.107	-0.022	-0.123	-0.204	1.196	0.318

BMI, Body Mass Index; FBG, Fasting Blood Glucose; FI, Fasting Insulin; FSH, Follicle stimulating hormone; LH, Leutinizing Hormone; TSH, Thyroid stimulating hormone; TT=, Total Testosterone; TC, Total cholesterole; TG, Triglycerides,HDLc, High Density Lipoprotein cholesterol; LDLc, Low Density lipoprotein cholesterol.

### 3.6 ANOVA for *CALP10* SNPs and PCOS

ANOVA (**Table 8**) confirmed the association of FSH (p=0.0001) and TC (p=0.09) with UCSNP-19; of FI (p=0.002) and TG (p=0.01) with UCSNP56; and of FBG (p=0.001), FI (p=0.004), FSH (p=0.02), and LDL-c (p=0.04) with UCSNP-63. However, the other SNPs were not associated with any of the covariates. The five SNPs in PCOS women were associated with higher counts for age (31.73 ± 5.62), weight (76.01 ± 10.26), and BMI (29.94 ± 3.96), and levels of FBG (6.01 ± 0.49), FI (17.17 ± 8.46), creatinine (57.67 ± 8.62), FSH (11.72 ± 6.65), LH (8.12 ± 4.74), TSH (2.51 ± 0.85), TT (2.14 ± 1.06), TC (5.50 ± 1.28), TG (2.48 ± 1.68), HDL-c (0.71 ± 0.25), and LDL-c (4.03 ± 1.32). Most of the high counts were confirmed in UCSNPs 19, 44, 56, and 66; these four SNPs were genotypically associated. Thus, among patients with PCOS, a potential relationship was documented with specific biochemical parameters, as well as with weight and BMI.

**Table 8:** One-way ANOVA analysis studied between SNPs in *CALP10* and PCOS covariates.

	*CALP10* (rs3842570)				*CALP10* (rs3792267)				*CALP10* (rs2975760)				*CALP10* (rs2975762)						*CALP10* (rs5030952)					
	II (n=14)	ID (n=40)	DD (n=36)	p Value	GG (n=60)	GA (n=24)	AA (n=06)	p Value	TT(n=55)	TC (n=26)	CC(n=09)	p Value	GG (n=29)		GA (n=25)		AA (n=36)	p Value	CC (n=68)		CT (n=15)		TT(n=07)	p Value
Age	30.86 ± 3.35	31.73 ± 5.62	29.75 ± 6.13	0.30	31.45 ± 4.93	29.75 ± 6.78	28.50 ± 6.25	0.26	31.02 ± 5.76	30.08 ± 5.53	31.56 ± 4.95	0.71	29.97 ± 6.61		30.60 ± 4.84		31.61 ± 5.18	0.49	30.76 ± 5.82		30.60 ± 5.33		31.57 ± 4.04	0.84
Weight	76.01 ± 10.26	73.75 ± 11.27	72.50 ± 12.26	0.62	73.32 ± 12.10	74.18 ± 10.33	74.13 ± 10.90	0.94	73.24 ± 12.00	73.73 ± 11.71	75.44 ± 7.52	0.86	73.33 ± 11.77		74.88 ± 8.35		72.93 ± 13.19	0.80	73.98 ± 11.48		73.54 ± 13.09		70.01 ± 7.79	0.42
BMI	29.89 ± 3.58	29.11 ± 5.10	28.61 ± 5.05	0.70	29.13 ± 5.03	29.02 ± 4.84	28.08 ± 3.41	0.88	28.87 ± 5.12	29.05 ± 4.63	29.94 ± 3.96	0.83	28.77 ± 4.09		29.50 ± 3.79		28.92 ± 6.02	0.84	29.23 ± 4.91		28.83 ± 5.49		27.53 ± 2.41	0.41
FBG	5.14 ± 1.27	4.96 ± 0.67	5.05 ± 0.93	0.78	5.01 ± 0.85	5.21 ± 1.01	4.46 ± 0.41	0.17	4.92 ± 0.77	5.28 ± 1.09	4.94 ± 0.79	0.22	4.94 ± 0.85		5.21 ± 1.08		4.96 ± 0.75	0.46	4.82 ± 0.75		5.49 ± 1.12		6.01 ± 0.49	0.001
FI	12.59 ± 7.60	9.89 ± 5.10	11.72 ± 6.65	0.26	11.43 ± 6.49	10.94 ± 5.85	6.74 ± 0.31	0.20	10.63 ± 6.16	11.88 ± 6.45	11.01 ± 6.07	0.70	9.28 ± 5.11		14.54 ± 7.91		9.97 ± 4.58	0.002	10.31 ± 5.17		11.41 ± 7.92		17.17 ± 8.46	0.004
Creatinine	55.50 ± 13.97	55.93 ± 14.64	49.97 ± 11.47	0.13	52.67 ± 14.85	55.83 ± 10.93	52.17 ± 7.11	0.61	54.25 ± 14.90	54.01 ± 10.55	47.22 ± 11.33	0.34	51.10 ± 8.94		55.04 ± 14.95		54.31 ± 15.43	0.50	53.06 ± 14.46		57.67 ± 8.62		48.57 ± 10.69	0.15
FSH	6.61 ± 2.64	6.70 ± 2.84	11.72 ± 6.65	0.0001	7.01 ± 3.28	6.42 ± 1.42	6.88 ± 1.60	0.68	6.82 ± 2.99	6.54 ± 2.31	7.86 ± 3.02	0.48	6.10 ± 1.75		7.39 ± 3.13		7.06 ± 3.18	0.20	6.88 ± 2.58		5.77 ± 1.42		8.77 ± 5.54	0.02
LH	6.13 ± 2.16	7.15 ± 4.63	8.12 ± 4.74	0.32	7.56 ± 4.62	6.82 ± 4.07	7.82 ± 3.92	0.76	7.66 ± 4.27	6.53 ± 4.39	8.06 ± 5.39	0.50	6.99 ± 4.09		7.60 ± 4.25		7.54 ± 4.84	0.84	7.57 ± 4.86		6.96 ± 2.17		6.41 ± 3.33	0.58
TSH	2.22 ± 0.99	2.30 ± 0.95	2.14 ± 0.68	0.72	2.19 ± 0.89	2.41 ± 0.80	1.82 ± 0.52	0.27	2.24 ± 0.82	2.33 ± 0.92	1.85 ± 0.80	0.34	2.51 ± 0.85		2.12 ± 0.76		2.07 ± 0.88	0.08	2.23 ± 0.88		2.27 ± 0.82		2.08 ± 0.65	0.76
TT	2.12 ± 0.76	2.08 ± 0.78	1.54 ± 0.94	0.01	1.82 ± 0.93	2.08 ± 0.79	1.53 ± 0.55	0.29	1.74 ± 0.83	2.13 ± 0.97	1.92 ± 0.78	0.17	1.76 ± 0.77		1.88 ± 0.87		1.95 ± 0.97	0.68	1.85 ± 0.91		1.82 ± 0.63		2.14 ± 1.06	0.46
TC	5.30 ± 1.20	5.25 ± 1.00	4.76 ± 1.11	0.09	5.21 ± 1.07	4.63 ± 1.05	5.28 ± 1.19	0.07	5.01 ± 1.15	5.04 ± 0.89	5.50 ± 1.28	0.45	5.33 ± 1.01		4.95 ± 1.40		4.93 ± 0.89	0.28	5.13 ± 1.16		4.87 ± 1.01		4.79 ± 0.28	0.39
TG	1.74 ± 1.16	1.93 ± 1.12	1.70 ± 1.11	0.65	1.90 ± 1.15	1.64 ± 1.15	1.59 ± 0.42	0.55	1.76 ± 1.01	1.93 ± 1.42	1.73 ± 0.83	0.80	2.27 ± 1.21		1.48 ± 1.02		1.66 ± 1.00	0.01	1.73 ± 1.03		1.85 ± 1.17		2.48 ± 1.68	0.06
HDLc	0.71 ± 0.25	0.60 ± 0.23	0.61 ± 0.21	0.27	0.64 ± 0.22	0.58 ± 0.25	0.58 ± 0.15	0.49	0.63 ± 0.24	0.60 ± 0.21	0.67 ± 0.19	0.70	0.59 ± 0.22		0.69 ± 0.29		0.60 ± 0.18	0.21	0.63 ± 0.22		0.65 ± 0.28		0.51 ± 0.17	0.11
LDLc	3.80 ± 1.13	3.77 ± 0.84	3.38 ± 0.93	0.14	3.71 ± 0.96	3.31 ± 0.79	3.98 ± 1.08	0.13	3.57 ± 0.95	3.57 ± 0.73	4.03 ± 1.32	0.37	3.70 ± 0.74		3.58 ± 1.18		3.58 ± 0.90	0.82	3.72 ± 0.97		3.38 ± 0.83		3.15 ± 0.47	0.04

BMI, Body Mass Index; FBG, Fasting Blood Glucose; FI, Fasting Insulin; FSH, Follicle stimulating hormone; LH, Leutinizing Hormone; TSH, Thyroid stimulating hormone; TT,Total Testosterone; TC, Total cholesterole; TG, Triglycerides; HDLc, High Density Lipoprotein cholesterol; LDLc, Low Density lipoprotein cholesterol.

### 3.7 Haplotype analysis for *CALP10* and PCOS

Haplotype analysis revealed a genetic association between SNPs in PCOS and non-PCOS women. The combination of D-A-T-G (p=0.04), D-G-C-A (p=0.01), D-A-T-A (p=0.01), I-G-C-G (p=0.04), D-G-C-G (p=0.02), I-A-T-G (p=0.03), I-A-C-A (p=0.01), D-A-C-A (p=0.006), I-A-C-G (p=0.01), and I-A-T-A (0.00) alleles showed association in PCOS women. In non-PCOS women, I-A-T-G (p=0.03), D-A-C-A (p=0.01), D-A-C-G (p=0.01), and I-A-T-A (0.00) showed a strong association. However, other SNPs were not associated (**Table 9**).

**Table 9:** Haplotype Analysis studied between PCOS and non-PCOS genotypes in 5 SNPs.

S. No	rs384250	rs3792267	rs2975760	rs2975767	Total	PCOS	Non-PCOS	Cumulative Frequency
1	D	G	T	G	0.2245	0.3062	0.1385	0.2245
2	I	G	T	G	0.2238	0.2973	0.1543	0.4483
3	D	G	T	A	0.1257	0.06	0.1848	0.5739
4	I	G	T	A	0.1133	0.1232	0.0983	0.6873
5	D	A	T	G	0.0559	0.0488	0.0637	0.7432
6	D	G	C	A	0.0502	0.0132	0.0719	0.7934
7	D	A	T	A	0.0447	0.0167	0.0823	0.8381
8	I	G	C	G	0.0339	0.0454	0	0.8721
9	D	G	C	G	0.0339	0.0268	0.0608	0.9059
10	I	A	T	G	0.0315	0.031	0.0337	0.9375
11	I	G	C	A	0.0308	NA	0.0915	0.9683
12	I	A	C	A	0.0111	0.0141	NA	0.9794
13	D	A	C	A	0.0102	0.0061	0.0102	0.9897
14	I	A	C	G	0.0054	0.0111	NA	0.9951
15	D	A	C	G	0.0049	NA	0.0101	1
16	I	A	T	A	0.0000	0.000	0.000	1

SNP5 (rs5030952) was used as reference.

### 3.8 Linkage disequilibrium analysis

**Figure 3:**
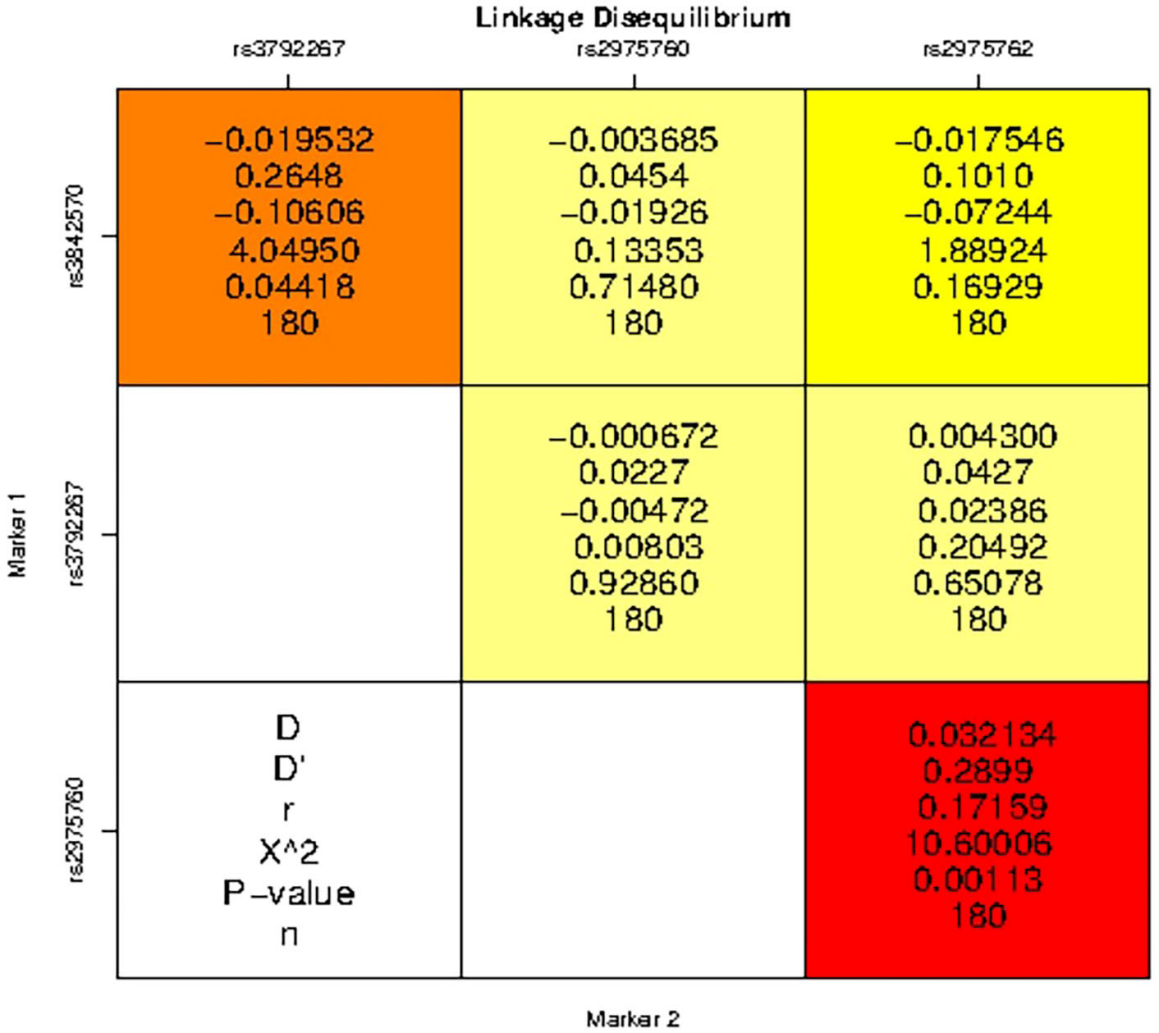
Linkage disequilibrium (LD) analysis studies between SNPs in CALP10 gene and PCOS disease risk.

**Figure 4:**
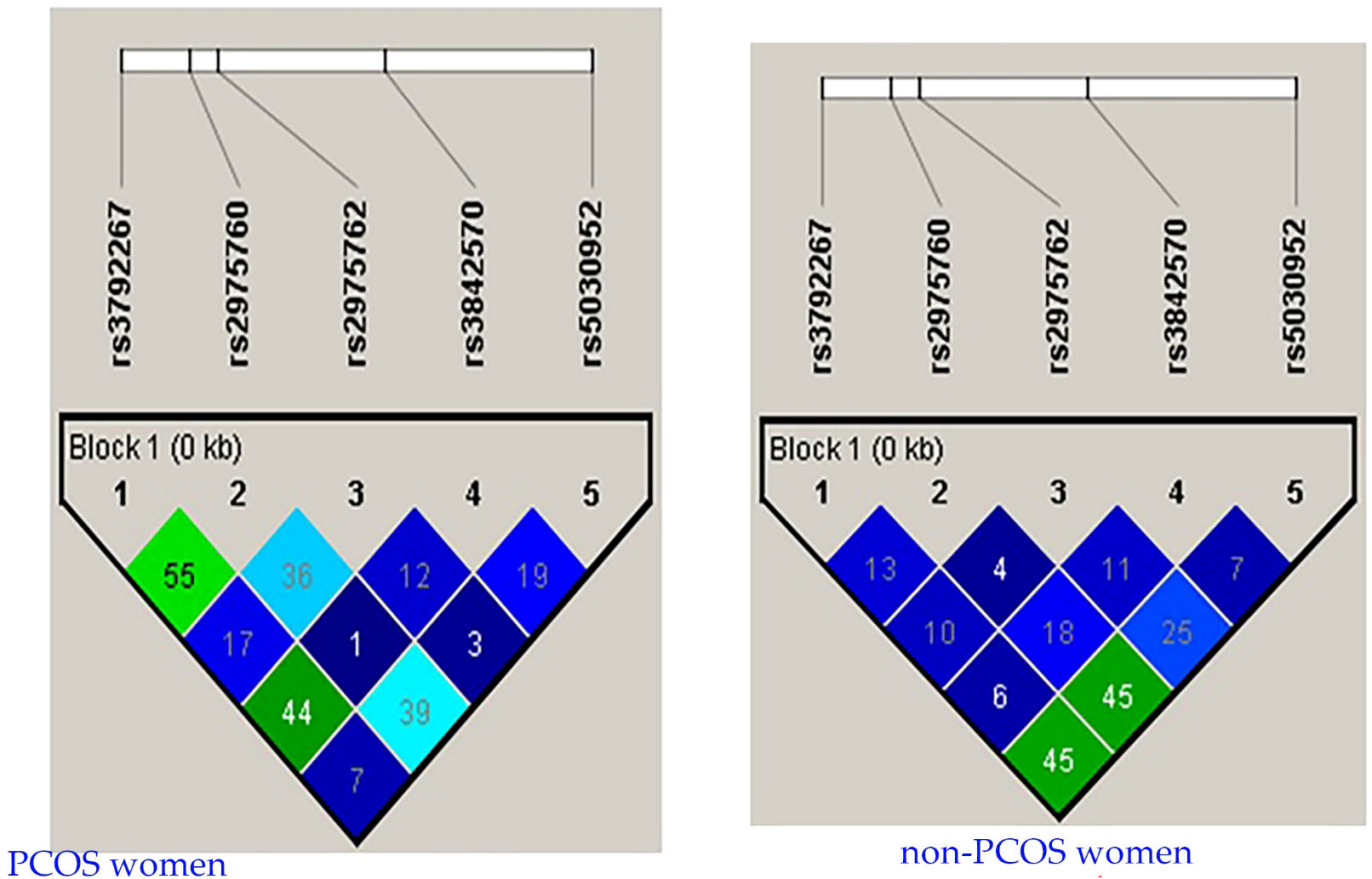
r^2^ pairwise measures of LD analysis between 5 SNPs in *CALP10* gene of Saudi women with PCOS and non-PCOS.

LD analysis revealed genetic associations between these five SNPs in women with PCOS. A strong association was detected between all combinations (**Table 10** and **Figure 3**), except for the combination of rs3972267 and rs5030952 in women without PCOS (p=0.058). Haplotype analysis of pairwise correlations closely mirrored the precise distance between SNPs. The combination of rs3972267 and rs5030952 in women without PCOS was an unsuitable predictor in CALP10 (**Figure 4**). The LD analysis confirmed that PCOS was associated and that CALP10 had a role, except for the combination of rs3972267 and rs5030952 in non-PCOS women.

**Table 10:** Linkage Disequilibrium analysis studied in Saudi women and 5 SNPs in *CALP10* gene.

Saudi Women	L1	L2	D’	r^2
PCOS Cases	rs3792267	rs2975760	0.554	0.025
PCOS Cases	rs3792267	rs2975762	0.17	0.008
PCOS Cases	rs3792267	rs3842570	0.442	0.03
PCOS Cases	rs3792267	rs5030952	0.077	0.0
PCOS Cases	rs2975760	rs2975762	0.36	0.036
PCOS Cases	rs2975760	rs3842570	0.014	0.0
PCOS Cases	rs2975760	rs5030952	0.391	0.01
PCOS Cases	rs2975762	rs3842570	0.123	0.011
PCOS Cases	rs2975762	rs5030952	0.033	0.0
PCOS Cases	rs3842570	rs5030952	0.198	0.012
Non-PCOS	rs3792267	rs2975760	0.135	0.017
Non-PCOS	rs3792267	rs2975762	0.106	0.005
Non-PCOS	rs3792267	rs3842570	0.065	0.001
Non-PCOS	rs3792267	rs5030952	0.459	0.058
Non-PCOS	rs2975760	rs2975762	0.04	0.001
Non-PCOS	rs2975760	rs3842570	0.182	0.004
Non-PCOS	rs2975760	rs5030952	0.454	0.063
Non-PCOS	rs2975762	rs3842570	0.118	0.004
Non-PCOS	rs2975762	rs5030952	0.255	0.009
Non-PCOS	rs3842570	rs5030952	0.077	0.0

PCOS, Polycystic ovary syndrome & Non-PCOS, Non-polyctsic ovary syndrome.

### 3.9 Generalized multifactor dimensionality reduction model analysis

#### 3.9.1 Gene–gene interaction analysis

**Table 11** describes the details of gene–gene interactions in women with PCOS. The GMDR analysis of gene–gene interactions indicated that the S1 model (rs5030952) and five-factor model, i.e., S1-S5 (rs5030952, rs2975760, rs3842570, rs32975762, rs5030952), had 10/10 cross-valid consistency and high-test accuracy (S1-S4, 0.9–0.667) when compared with the five-factor model (0.7278). The best model was the S1 gene–gene interaction model. The statistical test set in the S1 model (p<0.0001) was highly significant, followed by the two- and five-factor models of gene–gene interaction (p<0.05). This indicates a strong gene–gene interaction in the S1 model for PCOS risk among the five CALP10 SNPs.

**Table 11:** Determination of PCOS risk by gene-gene interaction analysis.

Model No	Best combination of genes	Training Accuracy	Testing Accuracy	CVC	P-value	Total Sensitivity	Total Specificity	X^2^	OR (95%CI)	F-Measure	Kappa
1	S1	0.9667	0.9667	10/10	<0.001	1.0	0.9667	157.5	Infinity	0.9677	0.9333
2	S1, S3	0.9673	0.9556	9/10	0.0001	1.0	0.9667	157.5	Infinity	0.9677	0.9333
3	S1-S3	0.9574	0.9278	8/10	0.0002	1.0	0.9222	154.1	Infinity	0.9222	0.9626
4	S1, S2, S4, S5	0.9185	0.9	6/10	0.0005	1.0	0.8333	128.5	Infinity	0.9231	0.8333
5	S1-S5	0.8383	0.7278	10/10	0.02	1.0	0.8722	10.6.7	Infinity	0.8867	0.7444

(S1= rs5030952; S2= rs2975760 S3= rs3842570 S4= rs32975762,S5=rs3792267).

#### 3.9.2 Dendrogram model

**Figure 5:**
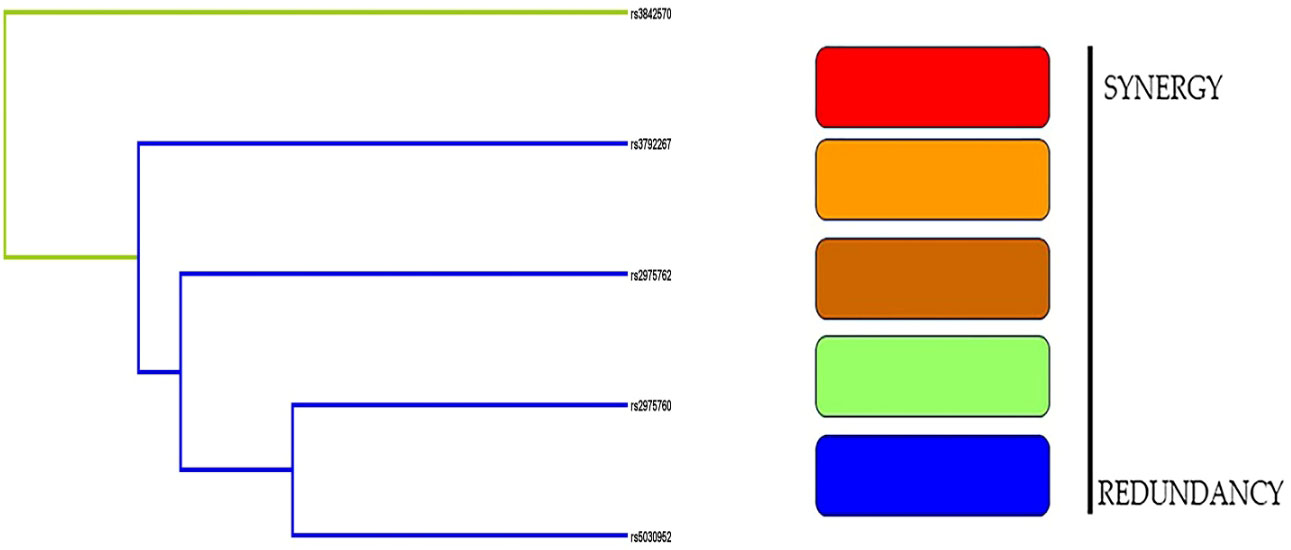
Dendogram analysis revealed the interaction between 5 SNPs and PCOS women.

In contrast to the empirical dendrogram presented in **Figure 5**, the dendrograms from randomly shuffled simulations were flat. rs3842570 had a green streak, whereas the remaining SNPs had blue streaks. The dendrogram analysis did not show a strong association in women with PCOS.

#### 3.9.3 Graphical depletion analysis

**Figure 6:**
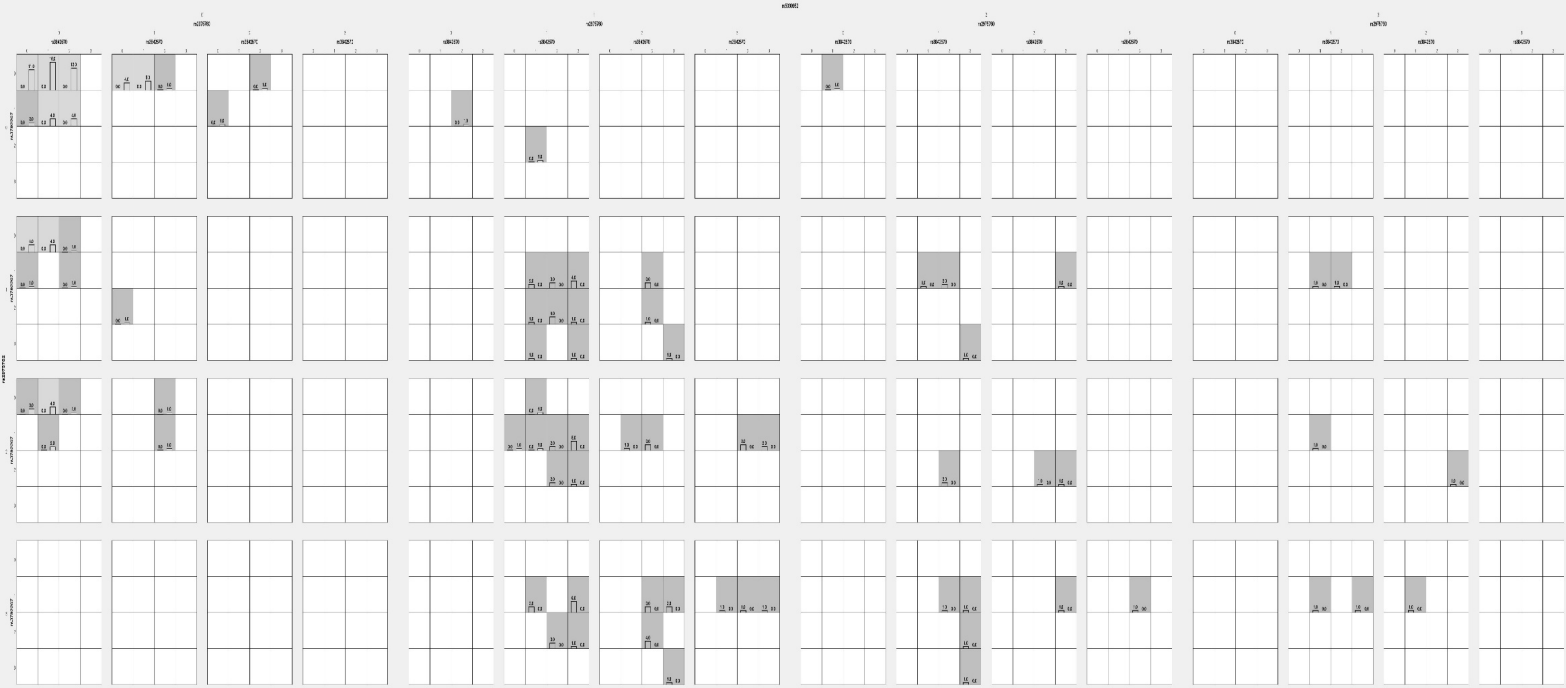
Analysis of Graphical depletion studied model using GMDR analysis in PCOS women with 5 SNPs.

The darker cells indicate a combination of high risks, and the light cells indicate low risks (**Figure 6**). A blank cell indicates the absence of genotype data. The bar confirms the hypothetical case on the left and the control on the right. The graphical depletion model showed only a limited high-risk combination in S1 between rs2975760 and rs3842570.

### 3.10 Classification of FBG levels according to BMI categorization in PCOS and non- PCOS women

The **Table 12**, in this study has categorized FBG levels according to different BMI categories such as Normal BMI (<24.9 kg/m^2^), Overweight (25.0-29.9 kg/m^2^), obesity (30.0-34.9 kg/m^2^) and morbid obesity (>35.0 kg/m^2^). When BMI levels measured between PCOS and non-PCOS, none of them were found to be associated in any of the classification (p<0.05). When it comes to FBG between PCOS and non-PCOS, FBG was associated between obesity (4.83 ± 0.66 vs 4.54 ± 0.53; p=0.001) and morbid-obesity (5.43 ± 1.55 vs 4.96 ± 0.88; p=0.01).

**Table 12:** Classification of FBG levels according to BMI categorization in PCOS and non-PCOS women.

BMI Levels	PCOS-BMI levels	Non-PCOS BMI Levels	P value	PCOS-FBG levels	Non-PCOS FBG levels	P Value
Normal BMI (<24.9 kg/m^2^)	22.22 ± 2.31	22.93 ± 4.71	0.38	4.89 ± 1.09	4.71 ± 0.56	0.16
Overweight (25.0-29.9 kg/m^2^)	27.82 ± 1.44	28.25 ± 1.30	0.33	5.14 ± 0.75	4.96 ± 0.86	0.13
Obesity (30.0-34.9 kg/m^2^)	32.39 ± 1.48	31.54 ± 4.54	0.09	4.83 ± 0.66	4.54 ± 0.53	0.001
Morbid Obesity (>35.0 kg/m^2^)	38.24 ± 2.73	37.70 ± 2.37	0.15	5.43 ± 1.55	4.96 ± 0.88	0.01

PCOS, Polycystic ovary syndrome; Non-PCOS; Non-polyctsic ovary syndrome; FBG, Fasting Blood Gluose.

## 4 Discussion

An irregular or absent menstrual cycle is a common characteristic in women with PCOS. PCOS is defined as a hormonal condition in women that has a direct impact on the female reproductive system. The name PCOS is derived from cysts known to be filled with fluid in sacs that often develop in the ovaries, causing them to expand. Previous studies in the past have shown that approximately half of the women with PCOS become overweight or obese, indicating that BMI is involved in PCOS pathogenesis. Further, the co-occurrence of PCOS and obesity has a strong hereditary basis (28). PCOS studies in Saudi women are important because of the increasing prevalence of obesity, DM, and lifestyle diseases. PCOS is associated with an increased risk of impaired glucose tolerance, and variants of *CALP10* have been linked to IR. Therefore, *CALP10* SNPs were analyzed in Saudi women diagnosed with PCOS.

Among five SNPs studied, rs3792267 (UCSNP-43), rs2975760 (UCSNP-44), rs2975767 (UCSNP56), and rs5030952 (UCSNP-63) were associated with allele frequencies and genotype associations, including different genetic models. FBG was strongly associated with the MLR model. In ANOVA, FSH and TT in rs3842570; FI and TG in rs2975762; and FBG, FI, FSH, and LDL-c in rs5030952 were associated. Haplotype analysis revealed a positive association of allele combinations with PCOS, and LD analysis confirmed a positive association between the studied SNPs. No strong associations were found with gene–gene interactions, dendrograms, or graphical depletion models. However, a nominal association with genotype frequencies was documented in women with PCOS. Based on the results together, we state that these SNPs in *CALP10* might be associated with PCOS in women, in whom FBG and FI might play a role via IR.

Gupta et al. confirmed that UCSNP44 had the best SNP–SNP interaction. Here, we employed the MDR/GMDR statistical model to explore gene–gene interactions of five SNPs with PCOS (29). This analysis confirmed a normal association, limited by a low sample size, which was reflected by the dendrogram and graphical depletion methods.

Hormonal imbalance and metabolic dysregulation underlie PCOS pathogenesis (30). This disease is also characterized by problematic follicular development. Inflammation, congestion, and dystrophy were identified as the three probable PCOS pathologies by Fogue and Massabuau in 1910. According to the inflammatory theory, internal or external infection causes microcystic ovaries. According to the congestion theory, the lesion occurs as a result of a decrease in blood flow to the ovary caused by pressure or partial torsion. Finally, the dystrophy explanation proposes changes or aberrations in ovarian nutrition (31, 32). In 1953, Stein and Leventhal developed the Rotterdam Criteria to identify with PCOS women (33).

Multiple studies in Saudi women with PCOS have confirmed associations with various disorders. Aldossary et al. confirmed obesity as a risk factor for PCOS (34). Some studies dealt with the psychological burden among Saudi women with PCOS (35, 36). Another study confirmed low vitamin D levels in women with PCOS in Saudi Arabia (30). Low levels of vitamin D, FSH, sex hormone-binding globulin, and estradiol were recorded in women with PCOS, together with high androgen, LH, TT, FBG, TC, and LDLc levels (37).

**Figure 7:**
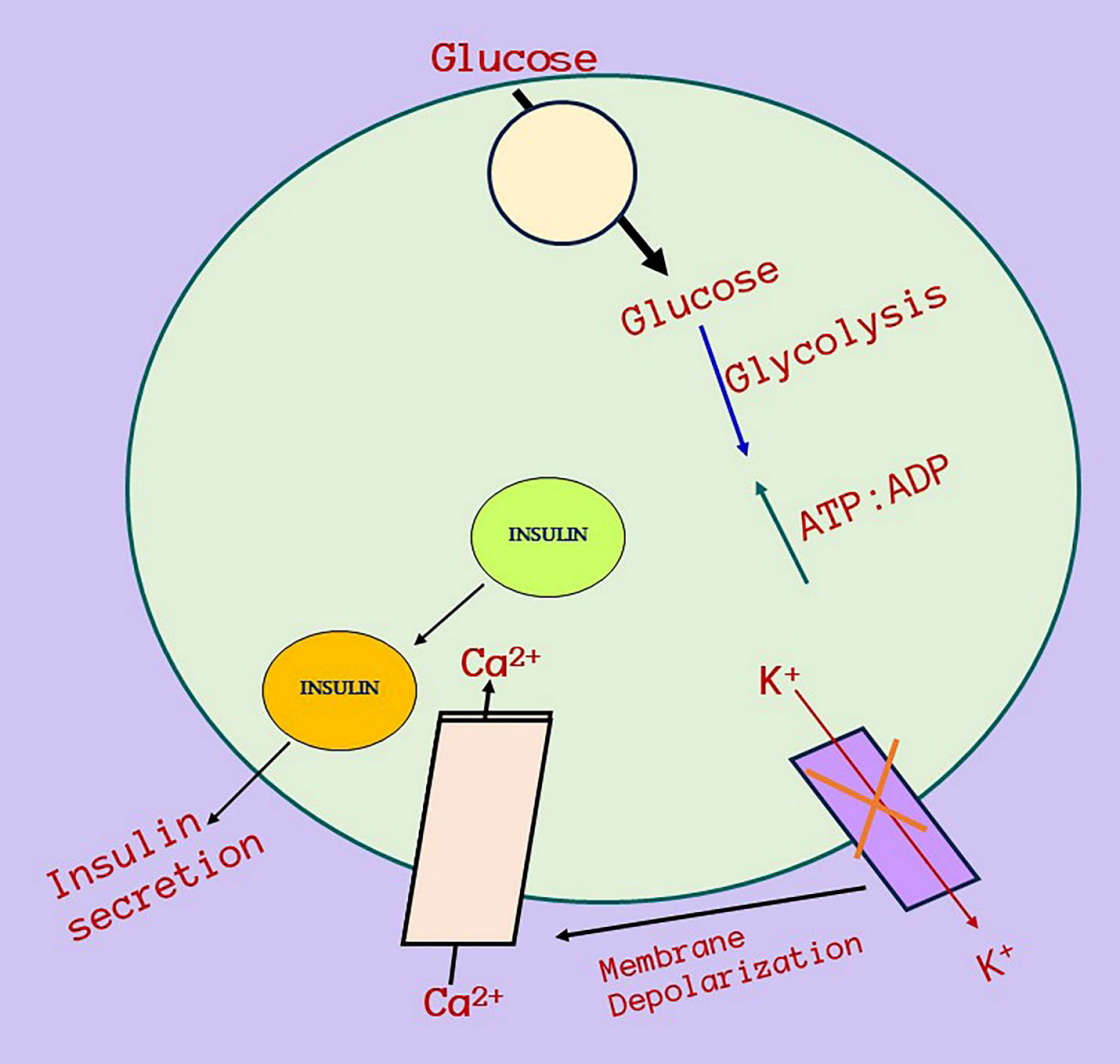
Relationship between *CALP10* gene, glucose metabolism, Insulin resistance and PCOS. Calpain-10 gene mechanism of action and its relationship with insulin resistance and then ultimately towards PCOS. This diagram depicts a potential strategy for Calpain-10 gene activation when glucose enters cells, increases the glucose absorption and metabolism raises ATP levels, which causes inactivation of ATP-sensitive K^+^ channels, membrane depolarization, and calcium influx and affects insulin and then insulin secretion. PCOS and diabetes is connected via insulin resistance and there is a high chances in the minimal of PCOS women to develop prediabetes in the future.

SNPs in *CALP10* are associated with the similar pathophysiology via Insulin resistance in both PCOS and T2DM (19). The encoded protein *(CALP10 or CAPN10)* is essential for calcium-regulated intracellular signaling. *CAPN10* was the first T2DM susceptibility gene to be positionally cloned (38). The gene contains 12–15 exons and is located on chromosome 2q37. It was associated with T2DM and is a possible gene for PCOS because IR and T2DM are associated with PCOS; variations in *CAPN10* can cause PCOS (39). **Figure 7** shows the relationship between *CALP10* and PCOS via glucose metabolism. IR and hyperinsulinemia affect 65–70% of women with PCOS, 70–80% obese women with PCOS, and 20–25% of lean women with PCOS (40). The link between T2DM and PCOS development may be due to shared insulin-related pathways. Patients who have IR are more likely to develop T2DM and cardiovascular disease (CVD) owing to the mutual interaction between IR and excess androgen levels. Continuous pancreatic stress under IR can result in impaired glucose tolerance and islet cell death, leading to T2DM (41). *CALP10* has been linked to the insulin signaling pathway; hence, SNPs in *CALP10* may determine insulin secretion and action (42).

Splicing governs the accurate removal of introns during mRNA processing. Introns contain splice sites, splicing regulatory sequences, as well as regulatory elements such as enhancers, silencers, and insulators, which control gene expression. Variants within these elements can affect the binding of transcription factors or other regulatory proteins, altering gene expression patterns. Dysregulation of gene expression underlies the development of various diseases, including cancer, autoimmune disorders, and metabolic conditions. Introns can also give rise to non-coding RNAs, which interact with other genes and proteins, influencing gene expression and cellular processes. Although several disease-associated genetic variants have been identified within introns, many intronic variants are benign and have no functional consequences. Researchers have employed various techniques such as functional studies, computational predictions, and large-scale association studies to evaluate their effects on gene function and disease susceptibility (43–45).

Here, we used four intronic SNPs and one 3′UTR SNP. rs3792267 was found to have no role in Saudi women with PCOS. This was supported by Anastasia et al. (46)Wiltgen et al. (47), Reddy et al. (48), and other studies (20, 38, 49–54). However, Marquez et al. found an association of rs3792267 in Chilean women with PCOS. Other SNPs in our study, such as rs3842570, were nominally associated with only a dominant model (55). This is supported by Vollmert et al. (38). However, other studies do not agree with our results (20, 47, 49–52, 55). In our study, the DD genotype was documented to be 40%, which is much higher than that reported by Dasgupta et al. and Unsal et al. (22.9% and 13.6%, respectively).

rs2975760 was associated with allele frequency, CC genotype, and dominant and recessive models, similar to studies by Al-Murshedi et al. (56) in Iraqi women and Yilmaz et al. (52) in Turkish women and women of other ethnicities. However, it was not associated with PCOS in other studies (20, 29, 38, 51, 54, 57, 58). Global studies indicate that rs2975760 is involved in IR, which is observed in PCOS and T2DM. rs2975760 has been studied in relation with PCOS, and different genotype frequencies have been documented. When it comes to our study, TT, TC, CC genotypes present in the PCOS women were 61.1%, 28.9% & 10%. The SNP rs2975760 studied in other global women confirmed differences in the 3 genotypes such as 61.8%, 20.7% & 17.4% in Dasgupta et al. studies, 75%, 25% & 0% in Unsal et al. studies, 58%, 27% & 15% in Al -Murshedi et al. studies and finally, 95.6%, 4.4% & 0% in Khazamipour et al. studies. Hence, the CC genotype plays an important role in specific ethnicities. rs2975760 in *CALP10* was studied in Saudi women with gestational diabetes mellitus, and no association was found (59 ). Our study findings were also in agreement with that study.

In our study, rs2975762 was strongly associated with allele frequency, the GA/AA genotype, and dominant and recessive models. The MAFs of the PCOS and non-PCOS groups were 53.9% and 23.3%, respectively. Vollmert et al. (38) reported MAFs of 44.7% in women with PCOS and 36.7% in controls. This SNP has also been documented in women of diverse ethnicities with PCOS.

The rs5030952 was the final SNP analyzed in our study. Statistical association between PCOS in women and allele frequencies, CT/TT genotypes, and the dominant model was confirmed. This SNP was not associated with other ethnic populations (20, 38, 47–52, 55). A recent review article confirmed the relationship between rs3842570, rs3792267, rs2975760, and rs5030952 SNPs of the *CALP10* gene and PCOS via the insulin resistance gene, which was confirmed as a risk factor, and these SNPs have been proven as possible causes for various phenotypes in PCOS women, affecting androgen production and causing hypercholesterolemia. As a result, specifically, rs3842570, and rs5030952 SNPs in metabolic or inflammatory pathways are regarded as genetic determinants for PCOS risk (60).

The only documented meta-analysis on PCOS with different SNPs confirmed that UCSNP-19, UCSNP-44, and UCSNP-63 were associated with PCOS, whereas UCSNP-43, UCSNP-56, UCSNP-58, and UCSNP-110 were not (21). Subgroup analysis revealed that UCSNP-19, UCSNP-45, and UCSNP-63 may lower the risk of PCOS in Asian women but not in Caucasian women. Allele frequencies and the dominant model were commonly associated with UCSNP-19, UCSNP-44, and UCSNP-63. The recessive model was associated only with UCSNP-44 and UCSNP-63. In our study, allele frequencies and dominant models were commonly associated with UCSNP-43, UCSNP-44, UCSNP56, UCSNP-63, and UCSNP-44; UCSNP56 was also associated with the recessive model. Heterozygous genotypes were commonly associated with UCSNP56 and UCSNP-63, and homozygous variant genotypes were commonly associated with UCSNP-44, UCSNP56, and UCSNP-63. Ben Salem et al. studied rs3792267, rs3842570, and rs5030952 in 127 Tunisian patients with PCOS and 150 control subjects, and the genotype analysis confirmed a non-significant association (61).

We assume that SNPs selected in *CALP10* gene may be playing a role in the PCOS women via Insulin resistance because in general 30-40% of PCOS women predicting to have elevated glucose levels which further tends to develop prediabetes or diabetes in the future. Apart from this, the prevalence of diabetes is high in Saudi women (62). The ANOVA analysis performed in this study showed FSH and TT levels were associated in rs3842570 SNP, fasting Insulin/Insulin (FI) and TG levels in rs2975760 SNP and FBG and Insulin levels in rs5030952 SNP. Based on the previous studies, PCOS is commonly associated with hormonal and metabolic abnormalities in which FSH, Insulin, TG and FBG levels are involved. Both FBG and TG levels are connected with insulin resistance and it is a common factor in the PCOS women. Additionally, PCOS is also involved with hyperandrogenism which indicates as elevated testosterone levels. Based on the obtained results from ANOVA analysis, we predict that rs3842570, rs2975760 and rs5030952 SNPs are playing a role with hyperandrogenism, hormonal and metabolic abnormalities in the PCOS women. The associated parameters in MLR and ANOVA analysis might be playing a role in PCOS women as these parameters are calculated using 5 SNPs present in the *CALP10* gene.

PCOS is maternally inherited with a 50% risk, in an autosomal dominant manner (63). Family history has a huge impact, especially in Saudi Arabia, where consanguineous marriages are common. Based on family history, it is essential to predict an individual’s risk for developing PCOS (64). In our study, 24.4% of the women had a family history of PCOS, and 33.3% had other family histories, including 16.7% with T2DM, 5.6% with hypertension (HTN), and 11.1% with a combination of T2DM and HTN. Among the control subjects, 28.9% of Saudi women had a family history of T2DM (14.4%), HTN (3.3%), or a combination thereof (11.1%). The remaining 71.1% of the control patients reported no disease in the family.

A cross-sectional questionnaire-based study among 710 women confirmed a PCOS prevalence of 32.5% in Madinah women of Saudi Arabia (65). The prevalence of this hormonal disorder has not been well reported in Saudi Arabia. Factors that affect post-diagnosis survival are as important as disease incidence in establishing prevalence. Documenting the prevalence of PCOS in Saudi Arabia is critical for healthcare planning, early detection and treatment, public awareness, research advancements, and policy progress. This can improve the circumstances of women with PCOS while promoting improved healthcare outcomes in the country.

Noncommunicable diseases (NCDs), including cancer, diabetes, CVD, and chronic respiratory diseases, are currently the primary cause of morbidity and mortality in Saudi Arabia, accounting for almost 78% of annual fatalities, with diabetes being the most prevalent. The emergence of multiple chronic medical conditions is known as multimorbidity (66). BMI has been demonstrated to be the leading risk factor in Saudi men and women, followed by high glucose levels as the second leading disease risk factor in Saudi women, and diabetes as the third leading risk factor in men. Obesity, HTN, and diabetes are growing problems in Saudi Arabia (67). By 2050, 1.3 billion cases of diabetes are predicted by 2050 (68). Besides, psychiatric disorders have increased between 30–46% in Saudi Arabia, and these diseases are more prevalent in confirmed chronic conditions, with women with PCOS having a higher risk of psychiatric problems, such as depression, anxiety, and stress (36). The combination of IR and β-cell dysfunction leads to the development of T2DM among women with PCOS, which is considered an endocrinological disorder and it includes obesity, HTN, dyslipidemia, and metabolic syndrome (22). The incidence of PCOS in women is increasing due to an aging population, and the possibility of metabolic syndrome is also increasing concomitantly (69). Women who experience PCOD during their reproductive years develop diabetes at around the age of 40 (70).

*CALP10* has been found to be the best predictor for assessing genetic SNPs in women with PCOS as *CALP10* is a validated marker for T2DM (7). In the present study, a positive association was found between UCSNPs 43, 44, 56, and 63. We showed that screening for SNPs in *CALP10* among women with PCOS in Saudi Arabia is a suitable approach for assessing current and future projected disorders in women diagnosed with PCOS.

Based on confirmation from gynecologists, PCOS and non-PCOS samples were collected, and clinical details such as acne, hirsutism, menstrual irregularities, infertility, and acanthosis nigricans were excluded, which is one of the limitations of this study. However, all women with PCOS were confirmed to have PCO, which is one of the strengths of this study. The selection of a two-digit sample size is another limitation. Performing Sanger sequencing for rs3792267, rs2975760, and rs2975762, and validating for rs5030952 was one of the strengths of this study. We did not document the diagnosis details of Rotterdam criteria, images of polycystic ovaries/missing the percentages of oligoanovulation and details of the infertility treatment in the PCOS women which will be considered as the third limitation of this study. The non-PCOS group had a family history of T2DM, HTN, or both. We selected non-PCOS women without any family history of PCOS, but we never checked or documented questions regarding other human diseases in the recruited women.

## 5 Conclusion

This study contributes to the understanding of the development of PCOS in Saudi women with rs3842570, rs2975760, rs2975767, and rs5030952 SNPs in *CALP10*. Additional statistics have also highlighted the risk factors for women with PCOS. This study recommends screening for additional SNPs present in *CALP10* with a larger sample size and conducting functional studies to validate the role of SNPs and specific pathways. The present study supports the hypothesis that SNPs in *CALP10* contribute to the susceptibility to PCOS in Saudi women. Replication studies with large sample sizes are warranted to verify the molecular role of PCOS in the global population. We strongly recommend conducting a nationwide study among women with PCOS to determine the prevalence of PCOS in Saudi Arabia.

## Data availability statement

The original contributions presented in the study are included in the article/supplementary material. Further inquiries can be directed to the corresponding author.

## Ethics statement

The studies involving humans were approved by Institutional Review Board at King Saud University. The studies were conducted in accordance with the local legislation and institutional requirements. The participants provided their written informed consent to participate in this study.

## Author contributions

AAA: Conceptualization, Data curation, Resources, Supervision, Writing – original draft. AFA: Conceptualization, Data curation, Formal analysis, Supervision, Writing – original draft, Writing – review & editing. IAK: Conceptualization, Data curation, Funding acquisition, Supervision, Writing – original draft, Writing – review & editing.
